# Supplementary material for: Visuospatial information foraging describes search behavior in learning latent environmental features
Source: Sci Rep. 2023 Jan 20;13:1126. doi: 10.1038/s41598-023-27662-9 (PMC9860038; doi:10.1038/s41598-023-27662-9)
Supplement: Supplementary file 1 — Supplementary Information. [file 41598_2023_27662_MOESM1_ESM.pdf]

Supplement

Visuospatial information foraging describes search behavior in learning latent environmental features

David L Barack[1*], Akram Bakkour[4], Daphna Shohamy[2], C Daniel Salzman[1,3]

[1]Department of Neuroscience, [2]Department of Psychology, and [3]Department of Psychiatry, Columbia University; [4]Department of Psychology, University of Chicago

* Corresponding author: dbarack@gmail.com

Results

While our results suggest that participants whose behavior can be better described as foraging were also faster at learning to reveal shapes, our analyses relied on a changepoint detection test (described in the methods). To validate our findings, we developed a second measure of learning (see supplemental methods below). We then ran the same analyses as reported in the main text for this alternative measure, confirming the findings reported in the main text.

We reasoned that a good measure of learning should account for differences across participants in the finalized level of performance. Some participants may perform the task with higher levels of performance, whereas others may accept a lower level of performance, but still have learned the shapes. For this reason, we rejected a single threshold level of performance to use a criterion for learning. In addition, different shapes might have been learned to different levels of stabilized performance. So, we also rejected any measures that aggregated across shapes. Finally, we reasoned that after learning, participants' performance should have stabilized at some level or other.

As an alternate measure of learning that possessed these three characteristics, we fit an exponential decay curve separately to each shape's learning curve for each participant (such as is depicted for the 'H' shape in Fig. 2A). This measure fits a different baseline for each shape, for each participant, and represents stabilized performance on the task. We then determined when the first trial for each shape dropped below that baseline, setting that as the end of learning for that shape. The end of learning for a participant was set to the last such changepoint across all shapes.

We next performed the same analyses reported in the main text but using the end of learning trial determined by the exponential decay method. We discovered that all three basic effects, of information foraging, of reward foraging, and of the correlation between foraging acuity and learning replicated on this analysis. The information outcome dropped below the average information outcome on the choice before a jump (Fig. S1A), just as was observed with the changepoint detection measure. In contrast, the reward outcome before a jump was not significantly below the average reward outcome (Fig. S1B; one-sample t-test, t(df=415) = -0.7559, $p > 0.45$), just as was observed with the changepoint detection measure. Finally, better information foraging scores for humans during learning predicted faster learning as determined by the exponential decay method (Fig. S1C; OLS, β_{slope} = -41.4383 ± 15.9604 trials/a.u. forager score, $p < 0.05$, ρ = -0.3839).

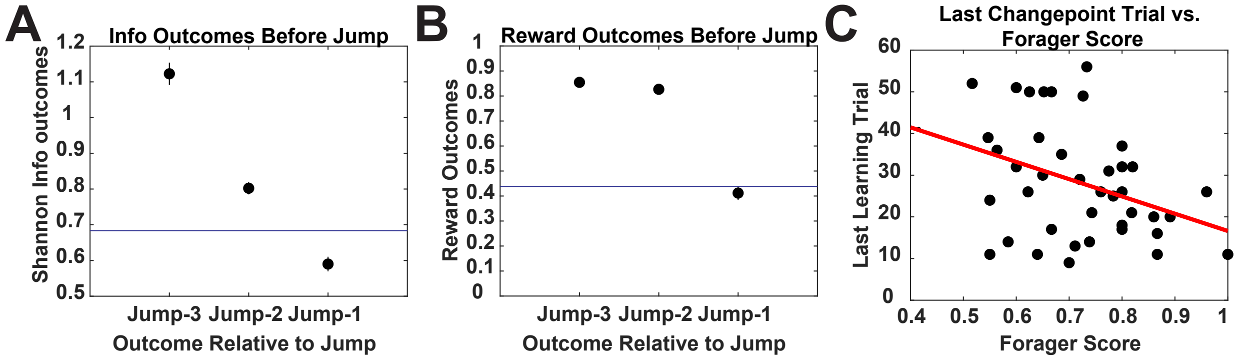

Supplementary Figure 1. A. *Information outcomes before a jump. The left panel depicts the three information outcomes before a jump. On the x-axis is the choice before the jump, on the y-axis is the information outcome, and the blue line is the average information outcome across choices during learning. Points = mean ± 1 s.e.m.; error bars sometimes occluded by data points.* ***B.*** *Reward outcomes before a jump. On the x-axis is the choice before the jump, on the y-axis is the reward outcome, and the blue line is the average reward outcome across choices during learning. Points = mean ± 1 s.e.m.; error bars sometimes occluded by data points.* ***C.*** *Information foraging correlates with speed of learning. On the x-axis is forager score, computed as described in the methods section, and on the y-axis is end of learning, as determined by the exponential decay threshold method.*

Methods

To compute the end of learning, we used an exponential decay method. We first calculated the number of choices required to finish each trial. We then grouped the trials by

shape, resulting in five vectors, one for each shape, where each element in the vector is the number of choices required to finish reveal the shape on a trial, with the first element = the first trial for that shape, and the last element = the last trial for that shape. These vectors correspond to learning curves (see Fig. 2A for an illustration of such a learning curve). We next fit an exponential decay function

$$y = \beta_1\left(e^{(\beta_2 * x)}\right) + \beta_3$$

for number of choices *y*, trial number *x*, shape parameter β_1, weight parameter β_2, and baseline threshold parameter β_3 to each such learning curve. For each shape separately, we determined the first trial that passed the baseline threshold, setting that as the end of learning for that shape. So long as a participant reached at least one such threshold for a shape they were included in the analysis. Only a single participant was ruled out by this analysis (because no trials ever reached the baseline threshold from the best-fit exponential for any of the five shapes).
